# Surgical Treatment and Proposed Modified Classification for Harrington Class III Periacetabular Metastases

**DOI:** 10.1111/os.12918

**Published:** 2021-03-04

**Authors:** Ran Wei, Chiao Yee Lim, Yi Yang, Xiao‐dong Tang, Tai‐qiang Yan, Rong‐li Yang, Wei Guo

**Affiliations:** ^1^ Musculoskeletal Tumor Center, Beijing Key Laboratory of Musculoskeletal Tumor Peking University People's Hospital Beijing China; ^2^ Department of Orthopaedic Surgery Kuala Lumpur Hospital Kuala Lumpur Malaysia

**Keywords:** Classification, Endoprosthesis, Periacetabular metastasis, Reconstruction, Surgery

## Abstract

**Objectives:**

This study aims to: (i) evaluate the outcome of patients with Harrington class III lesions who were treated according to Harrington classification; (ii) propose a modified surgical classification for Harrington class III lesions; and (iii) assess the efficiency of the proposed modified classification.

**Methods:**

This study composes two phases. During phase 1 (2006 to 2011), the clinical data of 16 patients with Harrington class III lesions who were treated by intralesional excision followed by reconstruction of antegrade/retrograde Steinmann pins/screws with cemented total hip arthroplasty (Harrington/modified Harrington procedure) were retrospectively reviewed and further analyzed synthetically to design a modified surgical classification system. In phase 2 (2013 to 2019), 62 patients with Harrington class III lesions were classified and surgically treated according to our modified classification. Functional outcome was assessed using the Musculoskeletal Tumor Society (MSTS) 93 scoring system. The outcome of local control was described using 2‐year recurrence‐free survival (RFS). Owing to the limited sample size, we considered *P* < 0.1 as significant.

**Results:**

In phase 1, the mean surgical time was 273.1 (180 to 390) min and the mean intraoperative hemorrhage was 2425.0 (400.0 to 8000.0) mL, respectively. The mean follow‐up time was 18.5 (2 to 54) months. Recurrence was found in 4 patients and the 2‐year RFS rate was 62.4% (95% confidence interval [*CI*] 31.6% to 93.2%). The mean postoperative MSTS93 score was 56.5% (20% to 90%). Based on the periacetabular bone destruction, we categorized the lesions into two subgroups: with the bone destruction distal to or around the inferior border of the sacroiliac joint (IIIa) and the bone destruction extended proximal to inferior border of the sacroiliac joint (IIIb). Six patients with IIIb lesions had significant prolonged surgical time (313.3 *vs* 249.0 min, *P* = 0.022), massive intraoperative hemorrhage (3533.3 *vs* 1760.0 mL, *P* = 0.093), poor functional outcome (46.7% *vs* 62.3%, *P* = 0.093), and unfavorable local control (31.3% *vs* 80.0%, *P* = 0.037) compared to the 10 patients with IIIa lesions. We then modified the surgical strategy for two subgroup of class III lesions: Harrington/modified Harrington procedure for IIIa lesions and en bloc resection followed by modular hemipelvic endoprosthesis replacement for IIIb lesions. Using the proposed modified surgical classification, 62 patients in the phase 2 study demonstrated improved surgical time (245.3 min, *P* = 0.086), intraoperative hemorrhage (1466.0 mL, *P* = 0.092), postoperative MSTS 93 scores (65.3%, *P* = 0.067), and 2‐year RFS rate (91.3%, *P* = 0.002) during a mean follow‐up time of 19.9 (1 to 60) months compared to those in the phase 1 study.

**Conclusion:**

The Harrington surgical classification is insufficient for class III lesions. We proposed modification of the classification for Harrington class III lesions by adding two subgroups and corresponding surgical strategies according to the involvement of bone destruction. Our proposed modified classification showed significant improvement in functional outcome and local control, along with acceptable surgical complexity in surgical management for Harrington class III lesions.

## Graphical abstract

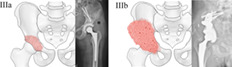

We propose a modified classification for Harrington class III periacetabular metastases by adding two subgroups and corresponding surgical strategies according to the involvement of bone destruction. The proposed classification showed significant improvement in functional outcome and local control, along with acceptable surgical complexity in surgical management for class III lesions.

## Introduction

The pelvis is the second most common site of metastatic bony malignancies after the spine^1^, ^2^, ^3^. The cornerstone of therapeutic strategy for pelvic metastasis is nonsurgical therapy, including protected weight‐bearing, radiotherapy, chemotherapy, and other adjuvant therapies^2^, ^4^. However, surgical treatment is often required for periacetabular metastatic lesions, owing to the severe pain and impaired ambulation, which cannot be relieved satisfactorily by conservative treatment^5^, ^6^, ^7^, ^8^.

The objectives of surgical treatment for periacetabular metastasis include prevention of pathological fractures and protrusion acetabuli, pain relief, and restoring ambulation and hip joint function^5^, ^6^, ^7^, ^8^. However, surgical strategies for periacetabular metastases remain diverse due to the various patterns of bone destruction^2^, ^4^, ^8^. For these purposes, Harrington proposed a surgical classification based on the acetabular insufficiency in 1981, which has enjoyed much popularity^1^, ^9^, ^10^. The Harrington classification categorizes periacetabular metastatic lesions into four classes with corresponding surgical strategies. Harrington class I lesions are contained cavitary defects, whereby the articular surfaces are disrupted but the walls and columns are intact. Intralesional excision is recommended, and conventional cemented total hip arthroplasty (THA) combined with metal mesh can achieve long‐term rigid fixation. In class II lesions, the medial wall and quadrilateral plates are deficient; however, the roof and acetabular rim are intact. The lesion is suggested to be intralesionally curetted and reconstructed using a flanged cup. In class III lesions, the medial wall, roof, and acetabular rim are deficient. Intralesional excision, acetabuloplasty of large Steinmann pins in cement followed by THA with a flanged cup, is recommended. Class IV lesions are solitary metastases, requiring en bloc resection and reconstruction with a reasonable anticipation of cure^11^, ^12^, ^13^.

Among the Harrington classification, class III lesions are the most challenging for surgical management because of the bone destruction, which may preclude solid reconstruction, and massive lesions, which may pose technical difficulties during excision of the tumor^14^. The description of class III lesions in Harrington classification remains ambiguous. Moreover, with the application of novel adjuvant therapeutics in past two decades, the life expectancy of patients with metastatic tumors has been prolonged dramatically. The implies that the intralesional curettage for periacetabular metastatic lesions in the Harrington classification would be insufficient to acquire consistent local control during patients’ survival period^7^, ^15^. Given that the local recurrence of bone metastasis is associated with severe pain and impaired function, surgery for periacetabular metastasis should also aim at achieving successful local control^16^, ^17^.

Thus, surgical classification and the corresponding surgical strategy for Harrington class III lesions should be reconsidered and modified. Although several modifications for surgical classification of Harrington class III lesions have been proposed, no consensus has been reached^5^, ^7^, ^9^, ^10^, ^18^, ^19^, ^20^, ^21^.

This study aims to: (i) evaluate the outcome of patients with Harrington class III lesions who were treated according to Harrington classification; (ii) propose a modified surgical classification for Harrington class III lesions; and (iii) assess the efficiency of the modified classification for treating patients with class III lesions. This study comprised two phases: a phase 1 study (2006 to 2011) aimed at reviewing the outcome of patients with Harrington class III lesions who were treated based on the original Harrington classification and proposing a modified classification for Harrington class III lesions; and a phase 2 study (2013 to 2019) aimed at evaluating the efficiency of the proposed modified classification for Harrington class III lesions.

## Materials and Methods

### *Inclusion and Exclusion Criteria*

The inclusion criteria were: (i) patients who were diagnosed with metastatic periacetabular metastasis; (ii) the lesion was classified as Harrington class III according to Harrington classification; (iii) patients who underwent surgeries in our center; and (iv) patients with full preoperative and postoperative clinical and radiological data.

The exclusion criteria were: (i) patients who received conservative treatments; (ii) incomplete medical records and radiographic data; and (iii) patients lost to follow‐up.

### *Patients*

This study composes two phases: phase 1 (2006 to 2011) and phase 2 (2013 to 2019).

In phase 1, we retrospectively reviewed 16 patients with Harrington class III lesions who were treated during January 2006 and December 2011 in our center. The last follow up of these patients was in June 2012. There were 7 men and 9 women, with a mean age of 52.6 (36 to 73) years. The primary malignancies were lung cancers (4), renal cancers (4), breast cancers (3), thyroid cancers (3), and others (2: endometrial cancer and an unknown cancer). (Table 1).

**TABLE 1:** Demographic characteristics of all patients

Factors		Total number/mean value	Phase 1	Phase 2	*P*
*N*		78	16	62	
Age (years)		56.0 (17 to 79)	52.6 (36 to 73)	56.9 (17 to 79)	0.22
Gender	Male	41	7	34	0.58
	Female	37	9	28	
Primary tumor site	Lung	26	4	22	0.35
	Kidney	12	4	8	
	Breast	8	3	5	
	Thyroid	6	3	3	
	Liver	4	0	4	
	Colon	3	0	3	
	Stomach	3	0	3	
	Bladder	2	0	2	
	Uterus	2	1	1	
	Malignant SFT	2	0	2	
	Others	5	0	5 (one each of prostate cancer, malignant pheochromocytoma, parotid gland carcinoma, GIST, and malignant thymoma)	
	Unknown	5	1	4	
Follow‐up time (months)		19.6 (1 to 60)	18.5 (2 to 54)	19.9 (1 to 60)	0.31

CI, confidence interval; GIST, gastrointestinal stromal tumor; MSTS 93, Musculoskeletal Tumor Society; RFS, recurrence‐free survival; SFT, solitary fibrous tumor.

In phase 2, 62 patients who were treated for Harrington class III lesions in our center between January 2013 to December 2019 were entered into this study phase. There were 34 men and 28 women, with an average age of 56.9 (17 to 79) years. The most common primary malignancy was lung cancer (22, 35.5%)(Table 1).

This study was carried out after obtaining approval from the hospital institutional review board committee.

### *Surgical Treatment*

The indications for surgery were severe pain unresponsive to conservative treatment, impaired ambulation, a primary tumor that was stable after sensitive therapies, and acceptable general condition.

Preoperatively, the periacetabular lesions and whole‐body metastatic status was assessed by imaging studies, including plain radiographs, CT, MRI, whole‐body bone scan, or positron emission tomography‐CT. For patients with hypervascular tumors (e.g. metastatic renal cancer, thyroid cancer, or liver cancer), preoperative selective artery embolization and/or intraoperative temporary occlusion of common iliac artery/aorta were performed to reduce intraoperative hemorrhage^22^.

**Fig. 1:**
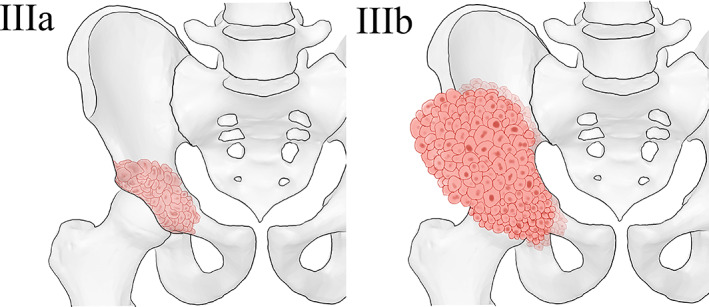
Proposed classification for Harrington class III lesions. IIIa: The bone destruction is distal to the inferior border of sacroiliac joint. IIIb: The bone destruction extends proximal to the inferior border of the sacroiliac joint.

In the phase 1 study, all 16 patients underwent intralesional excision followed by reconstruction of antegrade/retrograde Steinmann pins/screws with cemented THA (Harrington/modified Harrington procedure). The severity of bone destruction, surgical time, intraoperative hemorrhage, postoperative complications, and outcome of function and local control were reviewed and analyzed synthetically to design a modified classification system including class IIIa and IIIb lesions with corresponding surgical strategies for patients with Harrington class III lesions (Fig. 1). The design process and a detailed explanation of the modified classification will be described in the “Results.” In the phase 2 study, patients were first categorized into class IIIa and IIIb lesions. Patients with IIIa lesions consistently underwent Harrington/modified Harrington procedures (class IIIa surgery) (Fig. 3), while patients with IIIb lesions underwent en bloc resection followed by modular hemipelvic endoprosthesis replacement alone (Fig. 4) or combined with a screw‐rod system (Fig. 5) (class IIIb surgery).

Postoperative adjuvant therapies, including chemotherapy, endocrinal therapy, targeted therapy, and radiotherapy, were administered according to oncologists. The patients were assessed at the consultation every 3 months in the first year after surgery and every 6/12 months afterwards until death. Clinical and radiological assessments were performed at each visit to determine the outcomes for function and local control.

### *Outcome Measures*

The surgical time (min) and intraoperative hemorrhage (mL) were used to represent the complexity of different surgical procedures. Postoperative complications (such as dislocation and infection) were also recorded.

The overall survival (OS) was calculated from the date of surgery to death or to the date of the last follow up. The recurrence‐free survival (RFS) was calculated from the date of surgery to the date when the recurrence was diagnosed or to the date of the last follow‐up, and the 2‐year RFS rate was used to describe the outcome of local control.

#### *Musculoskeletal Tumor Society 93 Scoring System*

Functional outcomes were evaluated using the Musculoskeletal Tumor Society (MSTS) 93 scoring system for the lower extremity^23^. This system includes numerical values from 0 to 5 points assigned to each of the following six categories: pain, level of activity and restriction, emotional acceptance, use of orthopedic supports, walking ability, and gait. The final MSTS score is calculated as a percentage of the maximum possible score; the higher the percentage, the better the functional outcome.

### *Statistical Analysis*

Statistical analysis was performed using the Statistical Package for the Social Science (SPSS) software version 19.0 (IBM, Armonk, New York). Survival was calculated using the Kaplan–Meier method and was compared between different groups by log‐rank test. Nonparametric testing (Mann–Whitney *U*‐test) and Fisher's exact test were performed to compare the two groups and to determine statistical significance. Owing to the limited sample size, we considered *P* < 0.1 as significanct.

## Results

### *Clinical Outcome of Patients in Phase 1 Study*

All surgeries were performed uneventfully. The mean surgical time and intraoperative hemorrhage was 273.1 (180 to 390) min and 2425.0 (400.0 to 8000.0) mL, respectively. Two patients had postoperative complications: arterial embolism and venous thrombosis. The mean follow‐up time was 18.5 (2 to 54) months. Death occurred in 8 patients during the follow‐up period and the 2‐year overall survival (OS) rate was 49.7% (95% confidence interval [*CI*] 23.0% to 76.4%). Recurrence occurred in 4 patients and the 2‐year RFS rate was 62.4% (95% *CI*, 31.6% to 93.2%). The mean postoperative MSTS93 score was 56.5% (20% to 90%).

According to the severity of periacetabular bone destruction, we categorized the lesions into two subgroups: the bone destruction was distal to or around the inferior border of sacroiliac joint (IIIa), and the bone destruction extended proximal to inferior border of sacroiliac joint (IIIb) (Fig. 1).

**Fig. 2:**
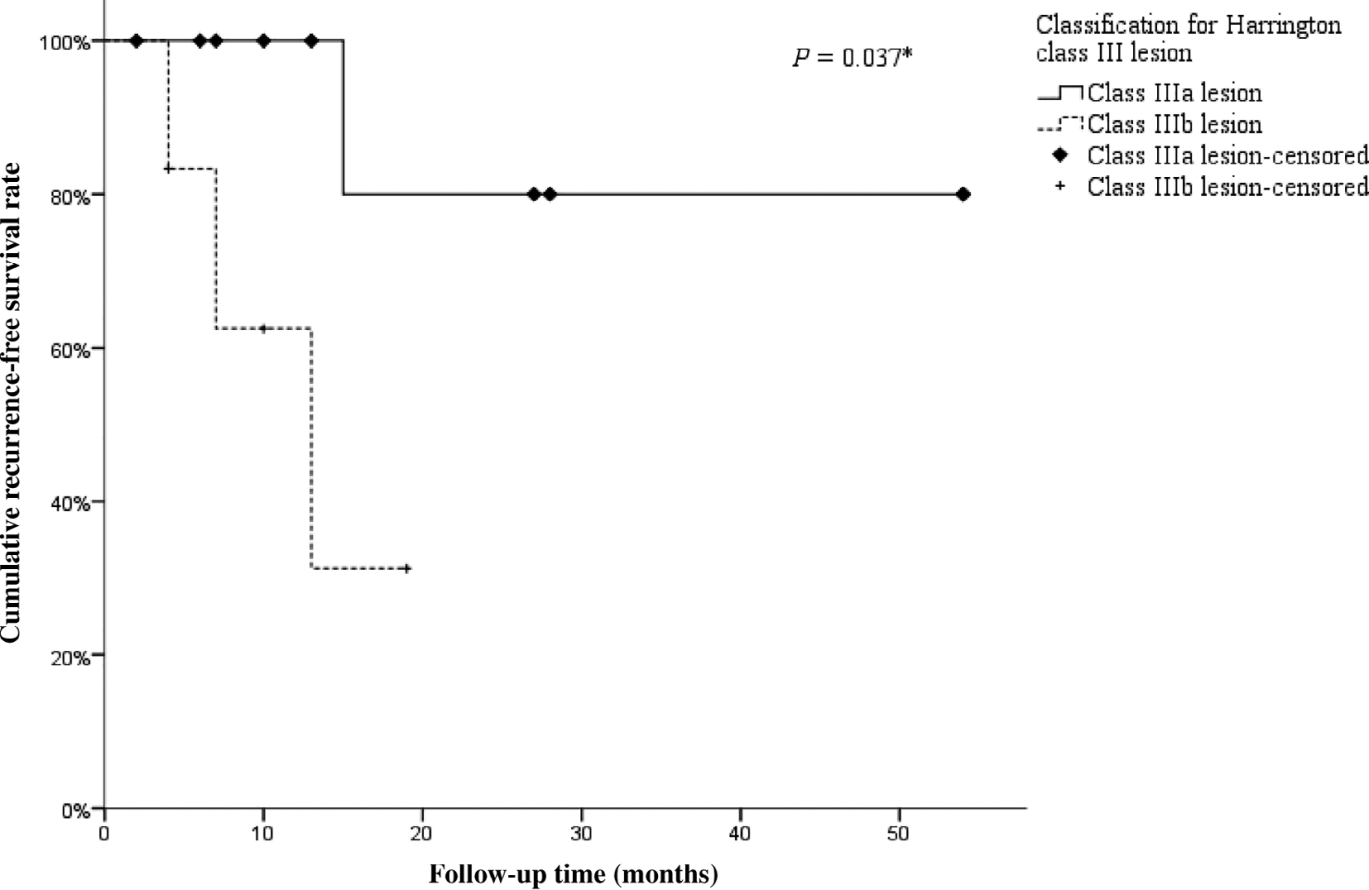
Recurrence‐free survival (RFS) of patients with different Harrington class III lesions in phase 1 study. After undergoing the Harrington/modified Harrington procedure, patients with class IIIb lesions showed significantly poorer local control compared to patients with class IIIa lesions.

Based on our classification for the periacetabular metastatic lesions, there were 10 patients with class IIIa lesions and 6 patients with class IIIb lesions. Patients with IIIb lesions showed significant prolonged surgical time and massive intraoperative hemorrhage compared to patients with IIIa lesions (surgical time: 313.3 *vs* 249.0 min, *P* = 0.022; intraoperative hemorrhage: 3533.3 *vs* 1760.0 mL, *P* = 0.093). The postoperative complication rate in patients with different types of lesions showed no significant difference (IIIa *vs* IIIb, 10.0% *vs* 16.7%, *P* = 0.63). By comparing the functional outcome, the MSTS93 scores of patients with IIIb lesions (46.7%) were significantly lower than those of patients with IIIa lesions (62.3%) (*P* = 0.093) (Table 2). Moreover, in the comparison of outcomes for local control, patients with IIIb lesions showed significantly lower 2‐year RFS rates compared to patients with IIIa lesions (31.3% *vs* 80.0%, *P* = 0.037) (Fig. 2).

**TABLE 2:** Clinical outcome of 16 patients with Harrington class III lesions in phase 1 study

Clinical characteristics	Mean/total	IIIa	IIIb	*P*
*N*	16	10	6	
Surgical time (min)	273.1 (180 to 390)	249.0 (180 to 390)	313.3 (270 to 370)	0.022*
Intraoperative hemorrhage (mL)	2425.0 (400.0 to 8000.0)	1760.0 (400.0 to 5000.0)	3533.3 (1400.0 to 8000.0)	0.093*
Postoperative complication rate (%)	12.5	10.0%	16.7	0.63
MSTS 93 score	56.5% (20.0% to 90.0%)	62.3% (46.7% to 90.0%)	46.7% (20.0% to 63.3%)	0.093*
2‐year RFS rate	62.4% (95% *CI* 31.6% to 93.2%)	80.0% (95% *CI* 44.9% to 100.0%)	31.3% (95% *CI* 0 to 79.3%)	0.037*

CI, confidence interval; MSTS 93, Musculoskeletal Tumor Society; RFS, recurrence‐free survival.

* Statistical significance.

### *Modified Surgical Classification for Harrington Class III Lesions*

**Fig. 3:**
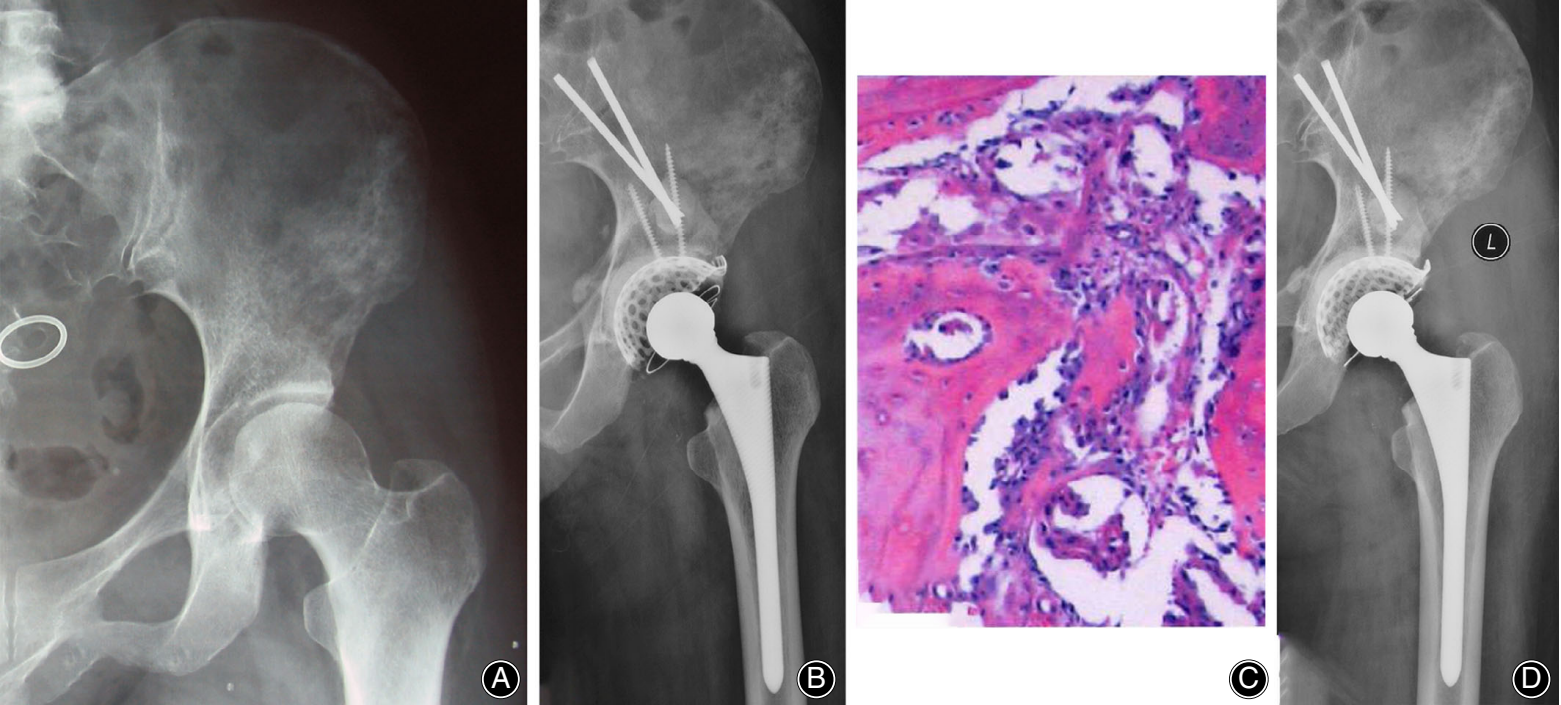
A 62‐year‐old female patient with metastatic periacetabular lung cancer presented with left‐sided class IIIa periacetabular lesion. (A) X‐ray showed that the bone destruction was distal to the inferior border of the sacroiliac joint. (B) The patient underwent intralesional excision followed by antegrade screws with cemented total hip arthroplasty (Harrington/modified Harrington procedure) (class IIIa surgery). (C) The patient was histologically diagnosed as metastatic lung adenocarcinoma. (D) X‐ray 6 months after surgery showed no evidence of tumor recurrence or mechanical endoprosthetic failure.

**Fig. 4:**
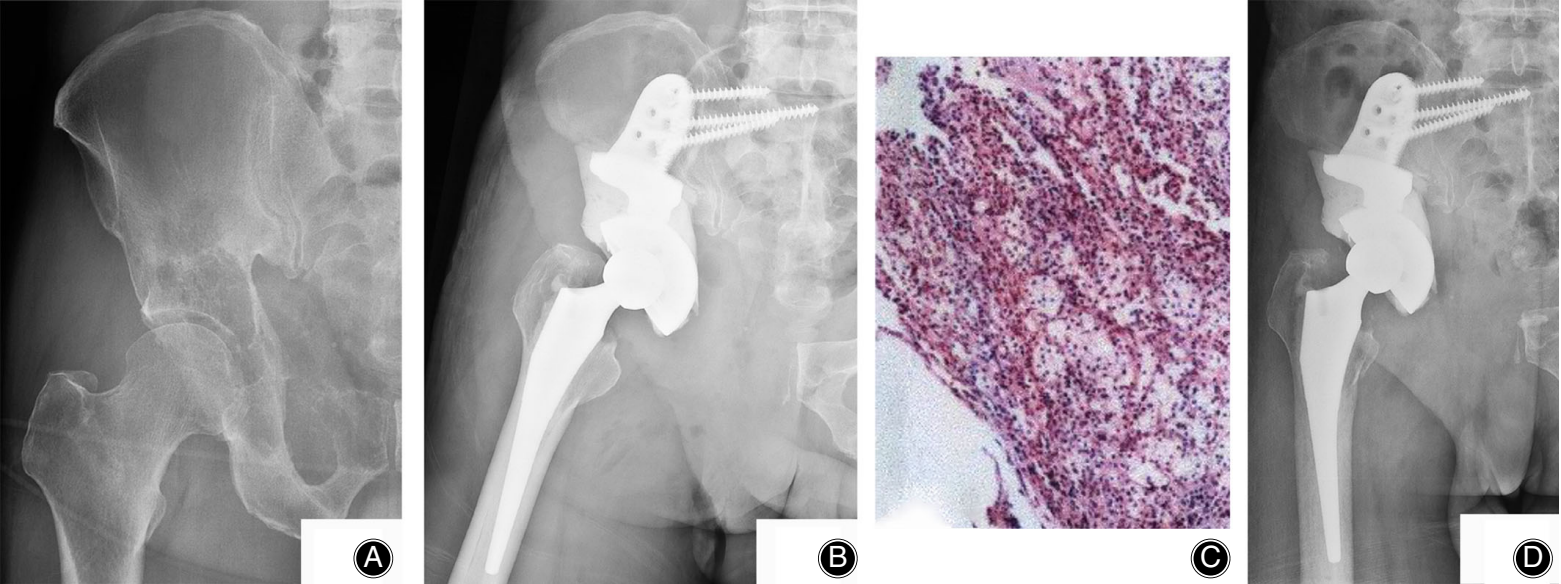
A 55‐year‐old male patient with metastatic renal cancer presented with right‐sided class IIIb periacetabular lesion. (A) X‐ray showed that the bone destruction extended proximal to the inferior border of the sacroiliac joint. (B) The patient underwent en bloc resection followed by modular hemipelvic endoprosthesis replacement (class IIIb surgery). (C) He was histologically diagnosed as having metastatic clear cell renal carcinoma. (D) X‐ray 12 months after surgery showed no evidence of tumor recurrence or mechanical endoprosthetic failure.

**Fig. 5:**
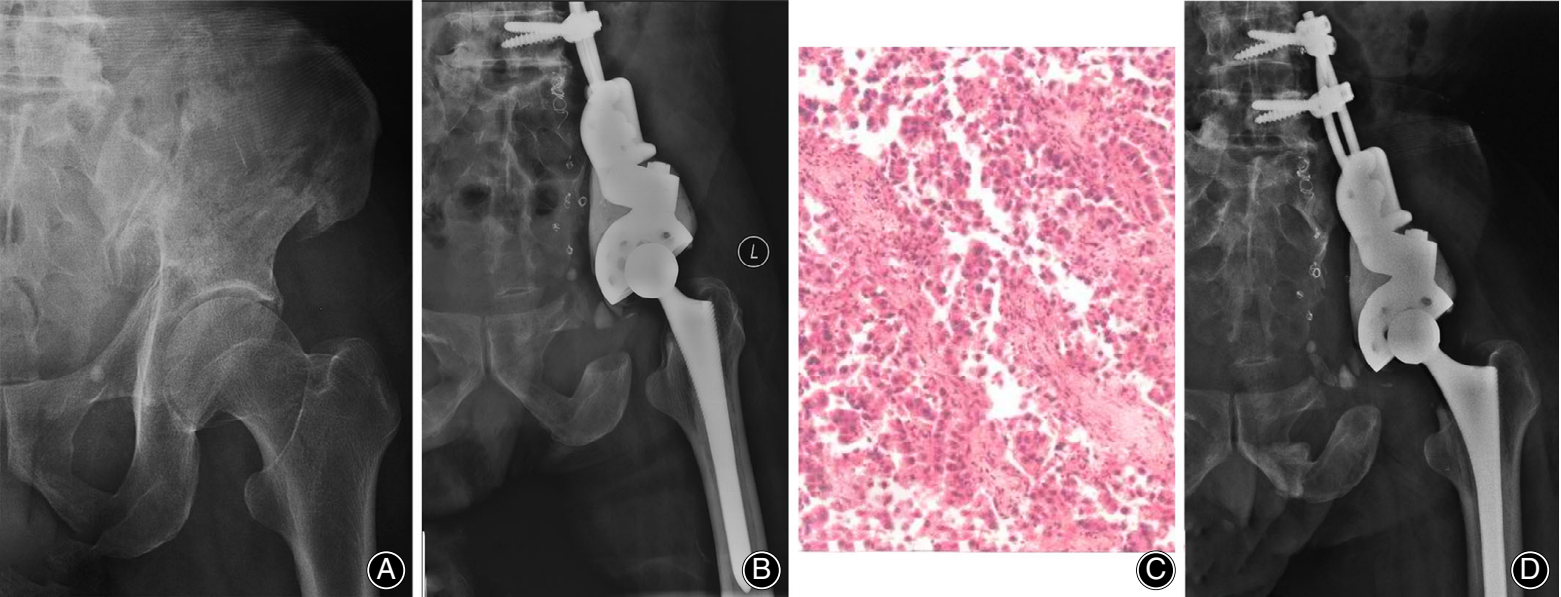
A 63‐year‐old male patient with metastatic renal cancer presented with left‐sided class IIIb periacetabular lesion. (A) X‐ray showed that the bone destruction extended proximal to the inferior border of the sacroiliac joint. (B) The patient underwent en bloc resection followed by modular hemipelvic endoprosthesis replacement combined with the screw–rod system (class IIIb surgery). (C) The patient was histologically diagnosed as having metastatic clear cell renal carcinoma. (D) X‐ray 14 months after surgery showed no evidence of tumor recurrence or mechanical endoprosthetic failure.

Based on the findings in the phase 1 study, the Harrington/modified Harrington procedure was demonstrated to be insufficient for Harrington class III lesions, particularly the lesions with massive periacetabular bone destruction, because of the prolonged surgical time, increased intraoperative blood loss, poor functional outcome, and unfavorable local control. Thus, we proposed a modified surgical classification for Harrington class III lesions: class IIIa lesions, which are the lesions with bone destruction distal to or around the inferior border of sacroiliac joint, should be treated by Harrington/modified Harrington procedures (class IIIa surgery) (illustrated in Fig. 3); class IIIb lesions, which are the lesions with bone destruction extended proximal to the inferior border of the sacroiliac joint, should be treated by en bloc resection followed by modular hemipelvic endoprosthesis replacement alone or combined with the screw–rod system (class IIIb surgery) (illustrated in Figs 4 & 5).

### *Clinical Outcome of Patients in Phase 2 Study*

All surgeries were completed successfully, with a mean surgical time of 245.3 (150 to 510) min and a mean intraoperative hemorrhage of 1466.0 (200 to 3600) mL. Postoperative complications occurred in 9 patients, including 5 patients with delayed wound healing, 3 patients with hip dislocation and 1 patient with venous thrombosis. The mean follow‐up time was 19.6 (1 to 60) months. A total of 39 patients (62.9%) died during the follow‐up period and the 2‐year OS rate was 45.9% (95% *CI* 32.4% to 59.4%). Recurrence occured in 3 patients (4.8%) and the 2‐year RFS rate was 91.3% (95% *CI* 81.9% to 100%). The postoperative MSTS 93 score was 65.3% (23.3% to 96.7%). (Table 3).

**TABLE 3:** Clinical outcome of patients with Harrington class III lesions in phase 2 study

Clinical characteristics	Mean/total	IIIa	IIIb	*P*
*N*	62	14	48	
Surgical time (min)	245.3 (150 to 510)	214.3 (160 to 300)	254.4 (150 to 510)	0.028*
Intraoperative hemorrhage (mL)	1466.0 (200 to 3600)	1260.0 (200 to 2500)	1526.0 (400 to 3600)	0.21
Postoperative complication rate (%)	14.5%	7.1	16.7	0.67
Postoperative MSTS 93 score (%)	65.3 (23.3 to 96.7)	68.6 (26.7 to 96.7)	64.3 (23.3 to 90.0)	0.41
2‐year RFS rate (%)	91.3 (95% *CI* 81.9 to 100.0)	83.3 (95% *CI* 53.5 to 100.0)	92.8 (95% *CI* 83.2 to 100.0)	0.43

CI, confidence interval; MSTS 93, Musculoskeletal Tumor Society; RFS, recurrence‐free survival.

* Statistical significance.

**TABLE 4:** Comparison of clinical outcome of patients with Harrington class III lesions in phase 1 and 2 studies

Clinical characteristics	Total number/mean value	Phase 1	Phase 2	*P*
*N*	78	16	62	
Surgical time (min)	251.0 (150 to 510)	273.1(180 to 390)	245.3 (150 to 510)	0.086*
Intraoperative hemorrhage (mL)	1662.7 (200 to 8000)	2425.0 (400 to 8000)	1466.0 (200 to 3600)	0.092*
Postoperative complication rate (%)	14.1	12.5	14.5	0.60
Postoperative MSTS 93 score (%)	63.5 (20.0 to 96.7)	56.5 (20.0 to 90.0)	65.3 (23.3 to 96.7)	0.067*
2‐year RFS rate (%)	86.0 (95% *CI* 76.0 to 96.0)	62.4 (95% *CI* 31.6 to 93.2)	91.3 (95% *CI* 81.9 to 100.0)	0.002*

CI, confidence interval; MSTS 93, Musculoskeletal Tumor Society; RFS, recurrence‐free survival.

* Statistical significance.

According to the modified classification for Harrington class III lesions, class IIIa and IIIb lesions were identified in 14 and 48 patients, respectively, and were subsequently treated by corresponding surgeries. Compared to patients who underwent class IIIa surgery, patients who underwent class IIIb surgery showed significantly prolonged surgical time (IIIa *vs* IIIb, 214.3 *vs* 254.4 min, *P* = 0.028), with, however, no significantly more intraoperative hemorrhage (IIIa *vs* IIIb, 1260.0 *vs* 1526.0, *P* = 0.21). Postoperative complication rates of patients with different classes of lesions demonstrated no significant difference (IIIa *vs* IIIb, 7.1% *vs* 16.7%, *P* = 0.67). Moreover, acceptable postoperative MSTS 93 scores were achieved in patients with both class IIIa and IIIb lesions (IIIa *vs* IIIb, 68.6% *vs* 64.3%, *P* = 0.41). In addition, the 2‐year RFS rates showed no significant difference between patients with class IIIa and IIIb lesions (IIIa *vs* IIIb, 83.3% *vs* 92.8%, *P* = 0.52).

### *Comparison of Clinical Outcome of Patients in Phase 1 and 2 Studies*

To evaluate the efficiency of the modified classification for Harrington class III lesions, we compared the clinical data of patients in the phase 1 study, who were treated according to the original Harrington classification, and patients in the phase 2 study, who were treated according to our proposed modified classification. The baseline data, including age, gender, primary tumor site, and follow‐up time, was first compared (Table 1). The results showed no significant difference, indicating that the clinical outcome of patients in the two phases of this study was comparable. Patients with Harrington class III lesions in the phase 2 study showed more preferred surgical time (245.3 *vs* 273.1 min, *P* = 0.086), intraoperative hemorrhage (1466.0 *vs* 2425.0 mL, *P* = 0.092), postoperative MSTS 93 scores (65.3% *vs* 56.5%, *P* = 0.067), and 2‐year RFS rate (91.3% *vs* 62.4%, *P* = 0.002) (Table 4).

## Discussion

### *Outcome of Patients with Harrington Class III Lesions Who Were Treated According to Harrington Classification*

The surgical strategy for Harrington class III lesions remains difficult and challenging^18^, ^19^. The class III lesions are defined in the Harrington classification as lesions with deficient medial wall, roof, and rim of the acetabulum, which indicates that the bone destruction could involve just the periacetabular bone or the whole ilium. The limited residual bone after removal of lesions with huge bone destruction (i.e. class IIIb lesions in our modified classification) precludes solid reconstruction, which may lead to an unfavorable functional outcome. In our phase 1 study, we performed the Harrington/modified Harrington procedure for all patients with class III lesions. Significantly poor MSTS 93 scores were illustrated in patients with class IIIb lesions. This indicated that the conventional and unified reconstruction procedure for Harrington class III lesions is insufficient to restore function.

We also found in the phase 1 study that surgical time and intraoperative hemorrhage were significantly elevated in patients with class IIIb lesions. This could be attributed to the intralesional curettage in the Harrington/modified Harrington procedure, which could be arduous and time‐consuming in class IIIb lesions. Moreover, patients with class IIIb lesions showed significantly poor local control. Given the improved survival of patients with malignant tumors arising from the application of novel adjuvant therapies, intralesional curettage in the Harrington classification for periacetabular metastasis should be altered to more aggressive procedures (like en bloc resection) to achieve continuous local control during patients’ survival period, particularly in class IIIb lesions^7^.

Concisely, we concluded in the phase 1 study that the Harrington classification and corresponding surgical strategy for class III lesions was insufficient. Although several works have focused on modifying the surgical strategy for Harrington class III lesions, there is no widely and formally accepted treatment algorithm and no study aimed at proposing a more specific classification for class III lesions (Table 5).

**TABLE 5:** Comparative studies focusing on surgical strategy for Harrington class III lesions

Authors	Year	Number of patients with class III lesions	Lesion removal methods	Reconstructive methods	Surgical time (min)	Intraoperative hemorrhage (mL)	Mean follow up/survival time (months)	Mean MSTS 93 socre (%)	Recurrence
Nilsson, *et al*.^9^	2000	30	Intralesional curettage	Harrington technique	181.7	2066.7	16.5	NR	NR
Marco, *et al*.^15^	2000	55	Intralesional curettage	Harrington/modified Harrington technique	290	2200	NR	NR	14
Benevenia, *et al*.^5^	2004	20	Intralesional curettage	Saddle endoprosthesis	180	1150	20	55.3	NR
Ho, *et al*.^7^	2010	35	Intralesional curettage	Modified Harrington technique	NR	NR	23.6	67	1
Bernthal, *et al*.^18^	2015	28	Intralesional curettage	Modified Harrington technique	NR	1200	17.7	NR	2
Kiatisevi, *et al*.^19^	2015	17	Intralesional curettage	Harrington/Modified Harrington technique	NR	2560	11	70	NR
Tsagozis, *et al*.^10^	2015	40	Intralesional curettage	Modified Harrington technique	NR	NR	12	NR	7
Current study (phase 2)		62	Intralesional curettage (IIIa) En bloc resection (IIIb)	Modified Harrington technique (IIIa); Modular hemipelvic endoprosthesis replacement (IIIb)	245.3	1466.0	19.9	65.3	3

MSTS 93, Musculoskeletal Tumor Society; NR, not recorded.

### *Modified Surgical Classification for Harrington Class III Lesions*

Based on the results of the phase 1 study, we hereby proposed a modified classification for Harrington class III lesions by defining class IIIa and IIIb lesions according to the severity of periacetabular bone destruction (Fig. 1). As for the surgical strategies for different lesions, given that the Harrington/modified Harrington procedure was demonstrated to be insufficient in surgical complexity, functional reconstruction, and local control for class IIIb lesions in phase 1 study, en bloc resection followed by modular hemipelvic endoprosthesis replacement alone or combined with the screw–rod system (class IIIb surgery) was assigned to class IIIb lesions, while the Harrington/modified Harrington procedure (class IIIa surgery) was assigned to class IIIa lesions. We subsequently conducted a phase 2 study, in which the patients were evaluated and treated as guided by our modified classification for Harrington class III lesions.

In phase 2 study, all patients showed acceptable postoperative MSTS 93 scores with no significant difference between the two subgroups. It may be attributed to using modular hemipelvic endoprosthesis, which has been proved to be adequate for reconstruction of massive pelvic bone defect after tumor removal, in the surgical strategy for class IIIb lesions in phase 2 study. Various endoprotheses have been applied in reconstruction after pelvic tumor resection, including saddle endoprostheses, pedestal or iliac stem endoprostheses, and modular hemipelvic endoprostheses^5^, ^12^, ^24^, ^25^. The functional outcome of using modular hemipelvic endoprosthesis in this study (64.3%) concurred with previous research regarding the same type of endoprosthesis (60.0% to 83.9%)^6^, ^25^, ^26^ and other types of endoprostheses (47.0% to 70.0%)^5^, ^12^, ^24^, ^27^.

Local recurrence causes relapse of pain and functional impairment *via* not only the recurrent lesion itself but also by inducing mechanical implant failure^18^. Good local control can improve patients’ quality of life continuously during the survival period. In the phase 2 study, the local control of patients with Harrington class III lesions was improved significantly by applying en bloc resection in selected patients guided by the proposed modified classification. This indicates that the proposed modified surgical classification for Harrington class III lesions could achieve desirable local control.

The complexity of the procedure is also a major concern in planning the surgery for periacetabular metastasis^8^, ^21^. Although class IIIb surgery is a more complex procedure than class IIIa surgery, demonstrated by significantly prolonged surgical time in the phase 2 study, the intraoperative hemorrhage of class IIIb surgery was increased. This may be because en bloc resection facilitates sufficient exposure of periacetabular lesions during surgery, which enables the surgeons to directly identify and ligate the tumor blood vessels. Therefore, the massive bleeding during intralesional curettage for huge Harrington class III lesions could be avoided. Meanwhile, decreased intraoperative hemorrhage and a clear surgical field also facilitates completely removing the lesions, which may improve the local control to some extent.

### *Efficiency of the Modified Classification for Treating Patients with Harrington Class III Lesions*

To evaluate the efficiency of the proposed surgical classification for Harrington class III lesions, we compared the surgical complexity and the outcomes for function and local control between patients in two phases who were treated according to different surgical classifications. Compared to Harrington classification and its surgical strategy for class III lesions, the modified surgical classification reduced surgical time and intraoperative hemorrhage, and improved the postoperative MSTS 93 scores and 2‐year RFS rates significantly. Moreover, in comparison with previous studies, our modified classification shows desirable surgical complexity, postoperative functional outcome, and local control in patients with Harrington class III lesions, and specifically demonstrates the surgical strategy for class III lesions with massive periacetabular bone destruction (Table 5)^5^, ^7^, ^9^, ^10^, ^15^, ^18^, ^19^. Thus, our modified classification is efficient for planning surgeries for patients with Harrington class III lesions.

### *Limitations*

There were some limitations in our study. First, although it is the largest series to date of a modified surgical classification for Harrington class III lesions, the sample size of this study (particularly in phase 1) is still too small to delineate possible differences. This was the reason why we considered *P* < 0.1 instead of *P* < 0.05 as statistically significant in this study. Second, the uneven distribution of different classes of lesions in the phase 2 study may cast doubt on some results: most of the periacetabular metastatic lesions with mild bone destruction could be treated *via* non‐surgical approaches, while lesions that require surgical treatment are often characterized as extensive bone destruction. Collaborative efforts will be needed to increase the sample size of future studies.

## Conclusion

The Harrington surgical classification is insufficient for class III lesions. We propose modification of the classification for Harrington class III lesions by adding two subgroups and corresponding surgical strategies according to the involvement of bone destruction. Our proposed modified classification showed significant improvement in functional outcome and local control, along with acceptable surgical complexity in surgical management for Harrington class III lesions.

## References

[1] Harrington KD . The management of acetabular insufficiency secondary to metastatic malignant disease. J Bone Joint Surg Am, 1981, 63: 653–664. 6163784

[2] Issack PS , Kotwal SY , Lane JM . Management of metastatic bone disease of the acetabulum. J Am Acad Orthop Surg, 2013, 21: 685–695. 24187038 10.5435/JAAOS-21-11-685

[3] Shahid M , Saunders T , Jeys L , Grimer R . The outcome of surgical treatment for peri‐acetabular metastases. Bone Joint J, 2014, 96: 132–136. 24395324 10.1302/0301-620X.96B1.31571

[4] Hoshi M , Taguchi S , Takada J , Oebisu N , Nakamura H , Takami M . Palliative surgery for acetabular metastasis with pathological central dislocation of the hip joint after radiation therapy: a case report. Jpn J Clin Oncol, 2012, 42: 757–760. 22628609 10.1093/jjco/hys071

[5] Benevenia J , Cyran FP , Biermann JS , Patterson FR , Leeson MC . Treatment of advanced metastatic lesions of the acetabulum using the saddle prosthesis. Clin Orthop Relat Res, 2004, 426: 23–31. 10.1097/01.blo.0000141387.03035.3e 15346047

[6] Guo W , Li D , Tang X , Yang Y , Ji T . Reconstruction with modular hemipelvic prostheses for periacetabular tumor. Clin Orthop Relat Res, 2007, 461: 180–188. 17452921 10.1097/BLO.0b013e31806165d5

[7] Ho L , Ahlmann ER , Menendez LR . Modified Harrington reconstruction for advanced periacetabular metastatic disease. J Surg Oncol, 2010, 101: 170–174. 19937990 10.1002/jso.21440

[8] Ji T , Guo W , Yang RL , Tang S , Sun X . Clinical outcome and quality of life after surgery for peri‐acetabular metastases. J Bone Joint Surg Br, 2011, 93: 1104–1110. 21768637 10.1302/0301-620X.93B8.26155

[9] Nilsson J , Gustafson P , Fornander P , Ornstein E . The Harrington reconstruction for advanced periacetabular metastatic destruction: good outcome in 32 patients. Acta Orthop Scand, 2000, 71: 591–596. 11145386 10.1080/000164700317362226

[10] Tsagozis P , Wedin R , Brosjo O , Bauer H . Reconstruction of metastatic acetabular defects using a modified Harrington procedure. Acta Orthop, 2015, 86: 690–694. 26220078 10.3109/17453674.2015.1077308 PMC4750768

[11] Aboulafia AJ , Buch R , Mathews J , Li W , Malawer MM . Reconstruction using the saddle prosthesis following excision of primary and metastatic periacetabular tumors. Clin Orthop Relat Res, 1995, 314: 203–213. 7634637

[12] Bus MP , Szafranski A , Sellevold S , *et al*. LUMiC(R) Endoprosthetic reconstruction after Periacetabular tumor resection: short‐term results. Clin Orthop Relat Res, 2017, 475: 686–695. 27020434 10.1007/s11999-016-4805-4 PMC5289170

[13] Tang X , Guo W , Ji T . Reconstruction with modular hemipelvic prosthesis for the resection of solitary periacetabular metastasis. Arch Orthop Trauma Surg, 2011, 131: 1609–1615. 21915657 10.1007/s00402-011-1359-5

[14] Colman MW , Karim SM , Hirsch JA , et al. Percutaneous acetabuloplasty compared with open reconstruction for extensive periacetabular carcinoma metastases. J Arthroplasty, 2015, 30: 1586–1591. 26115981 10.1016/j.arth.2015.02.022

[15] Marco RA , Sheth DS , Boland PJ , Wunder JS , Siegel JA , Healey JH . Functional and oncological outcome of acetabular reconstruction for the treatment of metastatic disease. J Bone Joint Surg Am, 2000, 82: 642–651. 10819275 10.2106/00004623-200005000-00005

[16] Chataigner H , Onimus M . Surgery in spinal metastasis without spinal cord compression: indications and strategy related to the risk of recurrence. Eur Spine J, 2000, 9: 523–527. 11189921 10.1007/s005860000163 PMC3611410

[17] Tomita K , Kawahara N , Kobayashi T , Yoshida A , Murakami H , Akamaru T . Surgical strategy for spinal metastases. Spine (Phila Pa 1976), 2001, 26: 298–306. 11224867 10.1097/00007632-200102010-00016

[18] Bernthal NM , Price SL , Monument MJ , Wilkinson B , Jones KB , Randall RL . Outcomes of modified Harrington reconstructions for nonprimary periacetabular tumors: an effective and inexpensive technique. Ann Surg Oncol, 2015, 22: 3921–3928. 25777096 10.1245/s10434-015-4507-2

[19] Kiatisevi P , Sukunthanak B , Pakpianpairoj C , Liupolvanish P . Functional outcome and complications following reconstruction for Harrington class II and III periacetabular metastasis. World J Surg Oncol, 2015, 13: 4. 25578802 10.1186/1477-7819-13-4 PMC4326438

[20] Kunisada T , Choong PF . Major reconstruction for periacetabular metastasis: early complications and outcome following surgical treatment in 40 hips. Acta Orthop Scand, 2000, 71: 585–590. 11145385 10.1080/000164700317362217

[21] Tillman R , Tsuda Y , Puthiya Veettil M , *et al*. The long‐term outcomes of modified Harrington procedure using antegrade pins for periacetabular metastasis and haematological diseases. Bone Joint J, 2019, 101: 1557–1562. 31786990 10.1302/0301-620X.101B12.BJJ-2019-0265.R1

[22] Tang X , Guo W , Yang R , Tang S , Ji T . Evaluation of blood loss during limb salvage surgery for pelvic tumours. Int Orthop, 2009, 33: 751–756. 19089426 10.1007/s00264-008-0695-8 PMC2903120

[23] Enneking WF , Dunham W , Gebhardt MC , Malawar M . Pritchard DJ. A system for the functional evaluation of reconstructive procedures after surgical treatment of tumors of the musculoskeletal system. Clin Orthop Relat Res, 1993, 286: 241–246. 8425352

[24] De Paolis M , Biazzo A , Romagnoli C , Ali N , Giannini S , Donati DM . The use of iliac stem prosthesis for acetabular defects following resections for periacetabular tumors. Sci World J, 2013, 2013: 717031. 10.1155/2013/717031 PMC3819951 24250275

[25] Ji T , Yang Y , Tang X , Liang H , *et al*. 3D‐printed modular Hemipelvic Endoprosthetic reconstruction following Periacetabular tumor resection: early results of 80 consecutive cases. J Bone Joint Surg Am, 2020, 102: 1530–1541. 32427766 10.2106/JBJS.19.01437

[26] Ji T , Guo W , Yang RL , Tang XD , Wang YF . Modular hemipelvic endoprosthesis reconstruction‐‐experience in 100 patients with mid‐term follow‐up results. Eur J Surg Oncol, 2013, 39: 53–60. 23131428 10.1016/j.ejso.2012.10.002

[27] Jansen JA , van de Sande MA , Dijkstra PD . Poor long‐term clinical results of saddle prosthesis after resection of periacetabular tumors. Clin Orthop Relat Res, 2013, 471: 324–331. 23054524 10.1007/s11999-012-2631-x PMC3528941

